# 

**Published:** 1893-08-26

**Authors:** 


					SOS PIT A T \ August 26th, 1
~ W WITH EXTRA NURSING SUPPLEMENT..
. PRICE TWOPENCE. iT Tl Per Annum, 8s. 6d., post free.
?\ 361, Vol. XVI. Aug. 26th, 1893. JmC? - Monthly Parts, 6d. ; post free, 8d.
f&ktered at the General Post Office as a Newspaper for ]W?i Ditto per Annum, 8s., post free,
i "Emission in the United Kingdom and Abroad.]
No. 361. Vol. XIY. Saturday,
August 26th, 1893.
[Twopence.

				

